# Maternal Recognition of Pregnancy in the Horse: Are MicroRNAs the Secret Messengers?

**DOI:** 10.3390/ijms21020419

**Published:** 2020-01-09

**Authors:** Katrien Smits, Yannick Gansemans, Laurentijn Tilleman, Filip Van Nieuwerburgh, Margot Van De Velde, Ilse Gerits, Cyrillus Ververs, Kim Roels, Jan Govaere, Luc Peelman, Dieter Deforce, Ann Van Soom

**Affiliations:** 1 Department of Reproduction, Obstetrics and Herd Health, Faculty of Veterinary Medicine, Ghent University, Salisburylaan 133, 9820 Merelbeke, Belgium; 2 Laboratory of Pharmaceutical Biotechnology, Faculty of Pharmaceutical Sciences, Ghent University, Ottergemsesteenweg 460, 9000 Gent, Belgium; 3 Animal Genetics Lab, Faculty of Veterinary Medicine, Ghent University, Heidestraat 19, 9820 Merelbeke, Belgium

**Keywords:** maternal recognition of pregnancy, horse, RNA-Sequencing, proteomics, microRNA

## Abstract

The signal for maternal recognition of pregnancy (MRP) has still not been identified in the horse. High-throughput molecular biology at the embryo–maternal interface has substantially contributed to the knowledge on pathways affected during MRP, but an integrated study in which proteomics, transcriptomics and miRNA expression can be linked directly is currently lacking. The aim of this study was to provide such analysis. Endometrial biopsies, uterine fluid, embryonic tissues, and yolk sac fluid were collected 13 days after ovulation during pregnant and control cycles from the same mares. Micro-RNA-Sequencing was performed on all collected samples, mRNA-Sequencing on the same tissue samples and mass spectrometry was conducted previously on the same fluid samples. Differential expression of miRNA, mRNA and proteins showed high conformity with literature and confirmed involvement in pregnancy establishment, embryo quality, steroid synthesis and prostaglandin regulation, but the link between differential miRNAs and their targets was limited and did not indicate the identity of an unequivocal signal for MRP in the horse. Differential expression at the embryo–maternal interface was prominent, highlighting a potential role of miRNAs in embryo–maternal communication during early pregnancy in the horse. These data provide a strong basis for future targeted studies.

## 1. Introduction

Early pregnancy in the horse is characterized by several unique events: After selective transport through the utero-tubal junction, the equine embryo develops a glycoprotein capsule, remains spherical and moves throughout the uterus to signal its presence to the mare [1,2,3,4]. This embryonic signaling initiates a series of events leading to the persistence of the corpus luteum, progesterone production, and a receptive uterine environment to support the maintenance of gestation, a process known as maternal recognition of pregnancy (MRP) [5]. In the non-pregnant cycling mare, pulsatile release of prostaglandin F2α (PGF2α) causes degradation of the corpus luteum, resulting in a decline in progesterone. This process is triggered by endometrial upregulation of prostaglandin-endoperoxidase synthase 2 (PTGS2), the rate limiting enzyme in the biosynthesis of PGF2α, and enhanced by oxytocin through a positive feedback loop [6,7,8]. During pregnancy, the presence of the conceptus suppresses this mechanism; a transient repression of oxytocin receptor (OXTR) and PTGS2 is observed during days 11–15 of pregnancy [6,9,10,11,12,13]. Strikingly, the exact timing and nature of the primary embryo-derived MRP signal is still unknown in the horse, despite several decades of research on this topic [3,14]. In pigs and ruminants, the MRP signals are estrogens [15] and interferon tau [16] respectively, and while the equine embryo produces both estrogens and interferons, no convincing evidence exists for their signaling role in MRP [14]. Interestingly, the equine embryo also releases substantial amounts of prostaglandins (PGs) E2 and F2α; these PGs stimulate the uterine contractions which are responsible for embryo mobility [17]. This embryo mobility is on its turn crucial for MRP, as limiting the mobility by uterine ligation results in termination of the pregnancy [18]. Therefore, the embryo produces the very substance responsible for luteal regression, and even though embryo-derived PGs only act locally, a delicate balance and regulation exists to establish MRP and maintain pregnancy, highlighting the importance of the embryo–maternal dialogue [3].

While initial studies have focused on the identity of specific candidate signaling molecules, recent research on the topic of MRP in the horse has been broadened to all pathways involved in embryo–maternal communication around the timing of MRP. High throughput molecular biology at the embryo–maternal interface has substantially contributed to the knowledge on pathways affected during MRP. Technological advantages, including sequencing, favored development of transcriptomics and several studies assessed gene expression of the endometrium and the conceptus between days 8 and 16 of pregnancy [19,20,21,22,23,24]. While no differences between pregnant and cyclic mares were detected on day 8, analysis on days 12 and 14 indicated pregnancy related upregulation of genes involved in nutrient supply and endometrial receptivity and downregulation of trophoblast invasion factors [14]; by day 16, clear differences were established. More recent development of mass spectrometry allowing large-scale quantitative proteomics at the embryo–maternal interface highlighted the presence of prostaglandin F2 receptor inhibitor and a progesterone potentiating protein in equine blastocoel fluid on day 8 [25] and upregulation of several inhibitors of the prostaglandin synthesis in the uterine fluid (UF) of pregnant mares on day 13 [26]. 

Transcriptomics and proteomics provide complementary information, but mRNA abundances can only explain less than half of the variation in protein levels; the actual protein profile is influenced by post-transcriptional, translational and degradative regulation mechanisms [27]. Key players in these regulation mechanisms are the microRNAs (miRNAs). These well conserved non-coding RNAs bind with complementary sequences in the 3’ untranslated region of the target mRNA, resulting in gene silencing via translational repression or target degradation. By regulating gene expression, miRNAs are involved in most biological processes, including embryo development, pregnancy establishment, implantation, and placentation [28,29,30]. MiRNAs are present in several bodily fluids and in equine reproduction, they were first documented in the ovarian follicle, highlighting their role in cell communication during growth and maturation of the oocyte [31]. Recently, pregnancy related miRNA expression has been examined in the horse [32,33]. A potential signaling role during MRP was assessed first by quantitative PCR to examine differential expression of miRNAs in serum; this indicated pregnancy related expression of miRNAs targeting focal adhesion molecules and expression of selected target mRNAs and proteins was evaluated in the endometrium [34]. Recently, the same group assessed expression of non-coding RNAs in the equine endometrium, and even though 419 differentially expressed non-coding RNAs were found on day 11, no differential expression was detected on day 13 [35].

While all disciplines of molecular biology have contributed to the current knowledge on MRP in the horse, a holistic approach in which high-throughput proteomics, transcriptomics and miRNA expression at the embryo–maternal interface can be linked directly is currently lacking. The aim of this study was to provide such integrated analysis. All samples were taken 13 days after ovulation during pregnant and control cycles from the same mares. To assess a potential signaling role of miRNAs during MRP in the horse, miRNA-Sequencing was performed on the endometrium and UF from pregnant and control cycles and on the embryonic tissue and yolk sac (YS) fluid from the corresponding embryos. In order to directly link this miRNA expression to transcriptomics and proteomics, mRNA-Sequencing was conducted on the same endometrial and embryonic tissue samples and linked to mass spectrometry performed previously on the same UF and YS fluid samples [26]. The aim was to present both differential expression of miRNAs, mRNAs and proteins between pregnant and cyclic mares, as well as a larger scale overview of molecular aspects at the embryo–maternal dialogue around the timing of equine MRP.

## 2. Results

**Figure 1:**
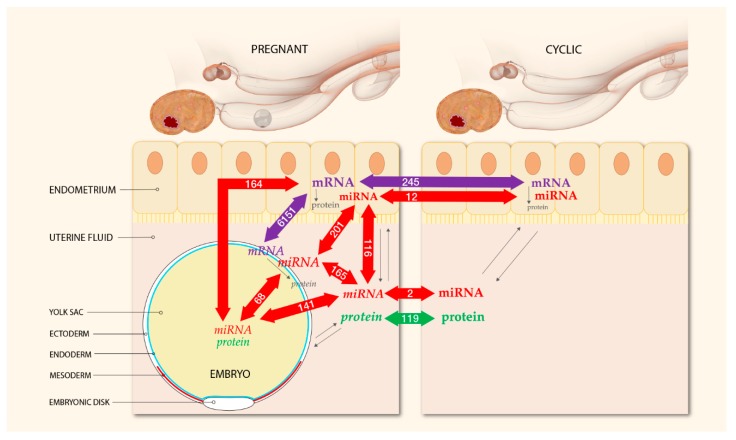
Experimental design and numbers of differentially expressed mRNAs, miRNAs and proteins detected at the embryo–maternal interface 13 days after ovulation in the horse. Micro-RNA-sequencing was performed of the uterine tissue and fluid of pregnant and cyclic mares and of the corresponding embryonic tissue and YS fluid. Messenger-RNA-Sequencing was conducted on the endometrium and the embryonic tissue of pregnant and cyclic mares, and proteomics of UF and YS fluid was reported previously [26]. All molecules originating from the embryo are displayed in *Italics*, and molecules of maternal origin are in **bold**. Colored arrows indicate the number of differentially expressed molecules in each comparison. Biological interactions are shown by black arrows; in all tissues mRNAs are translated into proteins and this interaction is influenced by miRNAs; molecules of both embryonic and maternal origin can be secreted into the UF and play a role in the embryo–maternal dialogue.

An overview of the experimental design including the total number of differentially expressed miRNAs, mRNAs, and proteins for each comparison in the endometrium and UF of pregnant and cyclic mares and in the YS fluid and the tissues of the corresponding embryos is presented in Figure 1. 

### 2.1. Messenger-RNA Sequencing

Out of the, on average, 60 million reads that were generated per sample, 95% were mapped to the Ensembl equine EquCab 2.80 reference genome. Among the 26,740 equine transcripts registered, 11,796 transcripts were considered quantifiable in the comparison between the pregnant endometrial biopsies and the cyclic endometrial biopsies. In the comparison between the pregnant endometrium and the embryonic tissues, a total of 11,602 equine transcripts were considered quantifiable.

A total of 245 genes were differentially expressed between the pregnant and the cyclic endometrium, with pregnancy-associated upregulation of 151 genes and downregulation of 94 genes (Figure 1, Supplementary File S1). 

**Figure 2:**
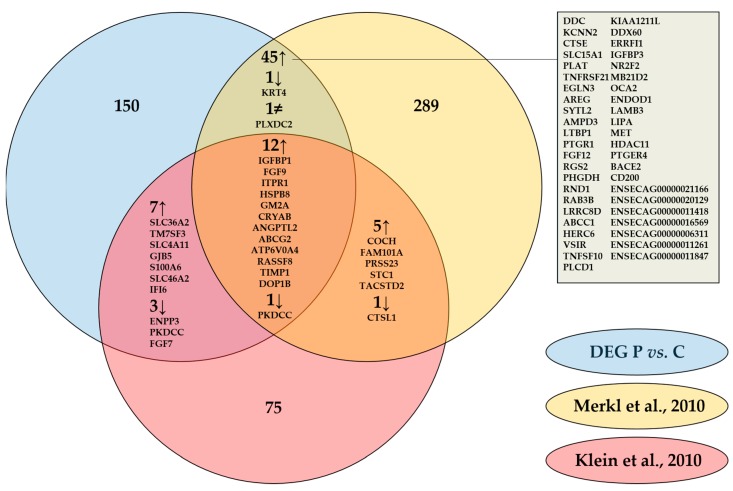
Comparison of differentially expressed genes (DEG) in equine endometrium of pregnant (P) versus cyclic (C) mares determined by RNA-Sequencing on day 13 in our study with analysis by microarray on day 12 by Merkl et al. [22] and on day 13.5 by Klein et al [19]. Gene IDs of commonly up-and downregulated genes are specified.

Our results coincided with literature as one third of the differentially expressed genes (DEGs) found were common with DEGs published previously by Klein et al. [19] (Gene symbols) and by Merkl et al. [22] (Ensembl Gene IDs), who compared endometrial gene expression of pregnant and cyclic mares by microarray on day 13.5 and on day 12 respectively (Figure 2). Common pregnancy associated upregulation with both studies was detected for 12 genes, with Merkl et al. [22] specifically for another 45 genes, and with Klein et al. [19] for 7 genes. Three genes were commonly downregulated during pregnancy in our study and the one of Klein et al. [19], one was similar in our study and the one of Merkl et al. [22] and one was downregulated in all three studies. A discrepancy was only detected for one gene, plexin domain containing 2 (*PLXDC2*), which was found to be downregulated in our study and upregulated on day 12 [22].

Assessment of differential gene expression in the pregnant endometrium and the corresponding embryos showed 6151 DEGs, with 3128 upregulated in the embryo and 3023 genes upregulated in the endometrium (Supplementary File S2).

### 2.2. Micro-RNA Sequencing

Average raw read quality Phred scores were between 30 and 40 and read length distribution after 3’ miRNA adapter trimming and quality filtering (score > 20) showed a peak around 22 nucleotides, characteristic for miRNAs. Expression of 690 known mature miRNAs and 154 novel mature miRNAs was assessed, resulting in the identification of 362 known and 154 novel miRNAs across the samples. After filtering for null or low counts, a total of 386 miRNAs were subjected to differential expression in the endometrial biopsies (Supplementary File S3), and 305 in the UF samples (Supplementary File S4). Comparison of pregnant vs. cyclic mares revealed differential expression (FDR ≤ 0.05) of 8 known and 11 novel miRNAs in the endometrial biopsies and of 1 known and 5 novel miRNAs in the UF. However, the number of predicted novel miRNAs was likely to be overestimated with duplicates having quasi identical counts, fold change and FDR values, rather representing only a single or a few unique novels with multiple genome locations. After correction, the final result was downregulation of 2 known mature miRNAs (eca-miR-30c and eca-miR-144) in the pregnant vs. cyclic biopsies and upregulation of 6 known (eca-miR-130a, eca-miR-660, eca-miR-486-5p, eca-miR-132, eca-miR-362-5p, and eca-miR-221) and 4 novel miRNAs, corresponding to ppy-miR-613, mmu-miR-8092, hsa-miR-6131, and an unknown sequence (Figure 1). In the UF, pregnancy related upregulation of eca-miR-23b and downregulation of a novel miRNA corresponding to has-miR-1246 was found (Figure 1). 

While the number of differentially expressed miRNAs between pregnant and cyclic mares within the same tissue type was limited, assessment of differential expression between the different tissues and fluids at the embryo–maternal interface revealed a substantial number of differentially expressed miRNAs (Figure 1, Supplementary Files S8–S13).

### 2.3. Integrated Analysis of Proteomics, Transcriptomics, and Micro-RNA Expression

Comparison of proteomics and transcriptomics in pregnant vs. cyclic mares showed that for three genes which were upregulated in the pregnant endometrium, the corresponding protein was also upregulated in the UF of the pregnant mares, namely for prostaglandin reductase 1 (PTGR1), annexin 1 (ANXA1), and GM2 ganglioside activator (GM2A). For two genes which were upregulated in the pregnant endometrium, serine peptidase inhibitor (SPI2), and mannosidase beta (MANBA), the opposite was found with pregnancy-associated downregulation of the corresponding protein in the UF.

In order to gain insight into the origin of the differentially expressed (DE) proteins in the UF of pregnant vs. cyclic mares, a comparison was made with DE mRNAs in the embryo vs. pregnant endometrium. Seventy percent of the proteins in the UF which were upregulated during pregnancy were more likely to originate from the embryo as the corresponding mRNA was upregulated in the comparison of embryo vs. endometrium. For the pregnancy-associated downregulated UF proteins, the number of up-and downregulated mRNAs in the embryo vs. endometrium was similar. The identity of all genes and proteins is listed in Supplementary File S5. The three genes which were upregulated in the pregnant endometrium and for which the corresponding protein was also increased in the UF of pregnant mares were all expressed more in the endometrium than in the embryo, suggesting maternal origin.

**Figure 3:**
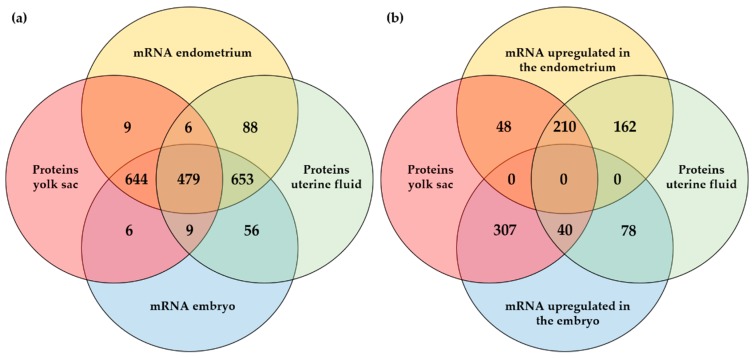
Comparison of proteins and mRNAs at the embryo–maternal interface on day 13 of pregnancy in the horse: (**a**) Comparison of mRNAs identified in the embryo and pregnant endometrium with the presence of the corresponding proteins in the uterine fluid (UF) and/or yolk sac (YS) fluid; (**b**) Comparison of differentially expressed mRNAs in the embryo vs. pregnant endometrium and the presence of the corresponding proteins in the UF and/or YS fluid.

To further explore the embryonic or maternal origin of proteins identified in the YS fluid and the UF during pregnancy, a comparison was made with the mRNAs identified in the pregnant endometrium and the embryo (Figure 3a) and with the DEGs in the embryo vs. endometrium (Figure 3b). For the proteins identified in the YS fluid and in the UF, the corresponding mRNAs were mostly found both in the embryo and in the endometrium. Translation into proteins showed an equal distribution between the UF and the YS fluid (Figure 3a). For the DEGs, half of the proteins for which the mRNA was upregulated in the endometrium, associated with maternal origin, was present in both the YS fluid and the UF, while the majority of proteins originating from the embryo was exclusively detected in the YS fluid (Figure 3b). The identities of all the gene and the protein numbers of Figure 3a,b are listed in Supplementary Files S6 and S7, respectively.

**Figure 4:**
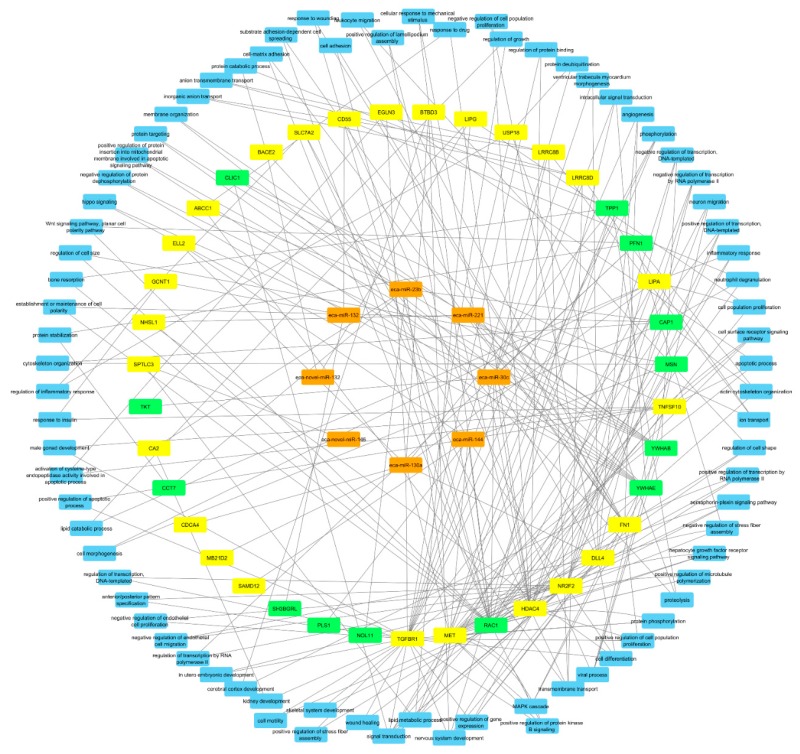
Interaction between differentially expressed miRNAs (orange), mRNAs (yellow) and proteins (green) in pregnant vs. cyclic mares 13 days after ovulation. Common Gene Ontology biological processes in which two or more molecules are involved are displayed in blue.

**Figure 5:**
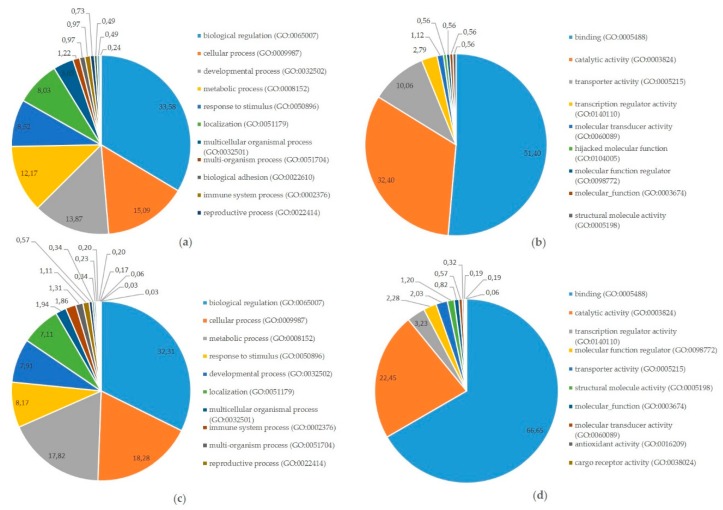
Involvement of targets of differentially expressed microRNAs at the equine embryo–maternal interface in Gene Ontology (GO) pathways: (**a**) Categorization in GO biological processes of proteins and mRNAs targeted by miRNAs differentially expressed in pregnant vs. cyclic horses as presented in (Table 1); (**b**) Categorization in GO Molecular functions of proteins and mRNAs targeted by miRNAs differentially expressed in pregnant vs. cyclic horses (Table 1); (**c**) Categorization in GO Biological processes of miRNA-mRNA target pairs differentially expressed between the embryo and the endometrium of pregnant mares; (**d**) Categorization in GO Molecular functions of miRNA-mRNA target pairs differentially expressed between the embryo and the endometrium of pregnant mares.

Finally, integrated analysis of differentially expressed miRNAs, mRNAs, and proteins in pregnant vs. cyclic mares was performed. Validated targets present in miRTarBase were assessed for all DE miRNAs. Targets present in miRTarBase for the DE miRNAs were subsequently compared with DE mRNAs and proteins found in our study. Targets of DE miRNAs in pregnant vs. cyclic endometrium corresponded with 27 DE endometrial mRNAs and 11 DE UF proteins, while targets of DE miRNAs in the UF of pregnant vs. cyclic mares corresponded with 3 DE endometrial mRNAs and 3 UF proteins (Table 1). Interaction and involvement of these targets in common Gene Ontology biological processes was visualized by Cytoscape 3.7.2 in Figure 4. Summary of all detected GO biological processes and molecular functions in which the mRNAs and proteins are involved is displayed in Figure 5a,b respectively.

Also the targets present in miRTarBase of the large number of differentially expressed miRNAs between the embryo and the endometrium were compared with differentially expressed mRNAs identified in this embryo–maternal comparison. Selection of consistent interactions with upregulation of a miRNA coinciding with downregulation of the target mRNA resulted in 274 miRNA-mRNA pairs (Supplementary File S14). Gene Ontology analysis summarizing involvement in biological processes and molecular functions is shown in Figure 5c,d.

## 3. Discussion

This study provides an integrated analysis of transcriptomics, proteomics and miRNA expression at the embryo–maternal interface around the signaling of maternal recognition of pregnancy in the horse. All samples were taken at day 13 after ovulation during the window of MRP signaling, which is estimated to occur between day 12 and day 14. All embryonic and maternal samples for the three different analyses (mRNA, proteins, miRNA) were taken at the same time from the same pregnant and control cycles in order to allow direct comparison of the results. For each mare, paired analysis of the pregnant and cyclic samples was performed to limit bias due to genetic variability. The first objective was to screen for candidate signaling molecules for MRP and to evaluate a potential role of miRNAs. Therefore, we focused on differential expression between pregnant and cyclic mares through horizontal comparison of mRNA and miRNA expression in the pregnant vs. cyclic endometrium and of protein and miRNA expression in the pregnant vs. cyclic UF. The second objective of the study was to gain general insight into the embryo–maternal interaction around equine MRP signaling by integrated vertical analysis of (differential) expression observed with different techniques in different tissues.

Transcriptomics of the equine endometrium showed differential expression of 245 genes. A similar approach has been published previously in the horse, using microarray or RNA-Sequencing to evaluate pregnancy associated endometrial gene expression between day 8 and day 18 [19,21,22,24,36]. Yet, another transcriptomics by RNA-Sequencing in our study provided added value as it allowed to build the bridge between proteomics and miRNA expression on day 13 and as RNA-Sequencing is more sensitive than microarray. High conformity with published results on day 12 [22] and day 13.5 [19] was found despite the use of other techniques, confirming both our methodology as well as the validity of these commonly found DEGs (Figure 2). Common upregulation in the endometrium of pregnant mares was found for several genes involved in key pathways at the embryo–maternal interface. This included upregulation of secretory molecules, namely insulin like growth factor binding protein 1 (IGFBP1), fibroblast growth factor 9 (FGF9), and TIMP metallopeptidase inhibitor 1 (TIMP1), which support embryonic development as well as endometrial proliferation and endometrial tissue remodeling during implantation [37,38,39]; IGFBP1 and TIMP1 have also been shown to inhibit trophoblast invasiveness. Common heat shock proteins were heat shock protein family B (small) member 8 (HSPB8) and crystallin alpha B (CRYAB), which are regulated by estrogens and involved in endometrial receptivity and implantation in human [40,41]. Also common upregulated genes involved in nutrient delivery through amino acid and lipid binding were found, namely GM2A, which was common in all three studies and solute carrier family 36 A (SLC36A), which was the most upregulated protein in the study of Klein et al. [19]. Furthermore, a role in pregnancy establishment through embryonic attachment, implantation or decidualization has been described in human and mice for amphiregulin (AREG) [42], ERBB receptor feedback inhibitor 1 (ERRFI1) [43], nuclear receptor subfamily 2 group F member 2 (NR2F2) [44] and prostaglandin E receptor 4 (PTGER4) [45]. 

Considering MRP, special interest goes to common pregnancy induced upregulation of genes involved in steroid hormone and prostaglandin regulation. In mice, ERRFI1 appeared crucial in progesterone mediated suppression of estrogen receptor 1 (ESR1) in the endometrium to prepare uterine receptivity [43]. Here, we detected upregulation of ERRFI1, but downregulation of ESR1 was not found yet, compliant with the results on day 12 [22]. In the horse, downregulation of ESR1 has been detected on day 13.5 [19] and day 15 [46] and is hypothesized to be involved in decreased oxytocin responsiveness, which on its turn could prevent luteal regression, crucial to MRP. Upregulation of FGF9 during pregnancy was highly significant. Its expression is induced by PGE2 and by estrogen and a recent study associated local PTGS2 enzymatic activity with FGF9 expression at the site of embryo implantation, highlighting a crucial role of FGF9 for the establishment and maintenance of pregnancy in mice [38,47]. The equine conceptus produces both estrogens and PGE2 [17], but their role as signaling factor for MRP has not been evidenced. Upregulation of PTGER4 and PTGR1 was found in the pregnant endometrium, coinciding with literature [22], but prostaglandin synthases were not affected.

Interestingly, not only PTGR1 mRNA was upregulated in the pregnant endometrium, also PTGR1 protein was more abundant in the UF of pregnant mares, compared to cycling control mares [36]. Another factor with an inhibitory effect on the prostaglandin synthesis, ANXA1, was also upregulated both at the mRNA and the protein level in the pregnant uterus. Also GM2A mRNA and protein was more highly expressed during pregnancy and has been linked to embryo and blastocyst quality in human [48]. However, despite these biologically interesting molecules, overall overlap between differentially expressed genes in the pregnant vs. cyclic endometrium and differentially expressed proteins in the pregnant vs. cyclic UF was very limited. This can be due to several aspects. In general, in mammalian cell lines mRNA abundances account for only 40% of protein concentrations which are further determined by post-transcriptional, translational and degradation regulations [27]. Moreover, the protein-per-mRNA ratios also depend on the biological role: housekeeping proteins and metabolic genes tend to be more stable than proteins involved in chromatin organization, transcriptional regulation. In our experimental set-up, we focused on proteins in the UF and as such, we aimed to avoid these stable housekeeping proteins and focus on signaling proteins, which are potentially produced and degraded more rapidly. Furthermore, we hypothesized that upregulated genes in the pregnant endometrium would be reflected by upregulation of the corresponding protein in the UF, but this might have been affected by both differences in timing and tissues. Upregulation of genes does not necessarily imply presence of the encoded protein at the same time and differential gene expression in the endometrial tissue appears not to coincide with secretion of the corresponding protein in the histotroph. Finally, proteins in the UF might not only be encoded by the pregnant endometrium, they might as well originate from the developing embryo. Indeed, comparison of the pregnancy induced proteins in the histotroph with differentially expressed genes between the embryo and the endometrium indicated embryonic origin of 70% of the proteins in the UF (Supplementary File S5). Yet, PTGR1, ANXA1, and GM2A, which were upregulated at the gene and protein level during pregnancy, were more expressed in the pregnant endometrium than in the corresponding embryo.

In order to gain insight into the role of microRNAs in posttranscriptional regulation and to assess a potential role of miRNAs as secret signaling molecules for MRP, miRNA-Sequencing was performed in all uterine and embryonic tissue and fluid samples (Figure 1, Table 1). Differential expression between pregnant and cyclic mares was found for 12 miRNAs in the endometrium and for two miRNAs in the UF. Research on the role of miRNAs during equine pregnancy is limited to a few studies focused on later stages of gestation [32,33], a recent study assessing non-coding RNAs in the endometrium in which no differences were found on day 13 [35] and one study determining differential expression of miRNAs in serum of pregnant vs. cyclic mares on day 9, day 11, and day 13 [34]. Two common miRNAs were detected. Coinciding with our findings, upregulation of miR-486-5p in pregnant vs. cyclic mares was also detected in whole blood at different time points during late pregnancy in the horse [32], but this miRNA was downregulated on day 9 in serum of pregnant vs. cyclic horses [34]. In human, downregulation of miR-486-5p has been associated with reproductive pathologies, including recurrent miscarriage [49] and polycystic ovary syndrome [50]. Expression of miR-30c was also opposite with downregulation in our study, while it was upregulated in serum of pregnant horses on day 13 [34]. Differences in results may arise from differences in tissue (endometrium vs. serum). Overall, differential miRNA expression was also variable amongst the serum samples taken at different days with no common differentially expressed miRNA throughout early pregnancy [34]. Small differences in timing may also influence the results, as Klohonatz et al. detected 419 differentially expressed non-coding RNAs in pregnant vs. cyclic endometrium on day 11 and none on day 13 [35]. In bovine, miR-30c has been associated with slow cleaving embryos of poor quality and addition of miR-30c to the embryo culture medium showed uptake by the bovine embryo resulting in increased apoptosis [51]. Other members of the miR-30 family, miR-30b and miR-30d, have been mostly associated with endometrial receptivity in human [52], but results are contradictory and upregulation of miR-30b was also found in the endometrium of patients with repeated implantation failure [53].

Differentially expressed miRNAs in pregnant vs. cyclic horses were also linked to pregnancy, steroid regulation and prostaglandin synthesis in other species. In conformity with our results, pregnancy associated upregulation of miR-132 was also detected in porcine endometrium, it was the only miRNA which was differentially expressed on day 12 in the pig [54]. Expression of miR-132 is associated with increased estradiol synthesis in mouse ovarian granulosa cells [55] and in human granulosa-like tumor cell lines [56], and might influence the process of MRP as such. Moreover, upregulation of miR-132-3p was found in follicular fluid yielding top quality embryos in human [57]. Coinciding with our results, upregulation during pregnancy was also found for miR-221 in bovine milk and for miR-23b in porcine endometrium and serum [58]. For miR-23b, ESR1 and beta-estradiol were identified among the top upstream regulators of the targets. MiR-130a [59], miR-362-3p [60], miR-221-3p [61], and miR-144 [62] have been examined in the context of human preeclampsia, a disorder linked with impaired migration and invasion of trophoblast cells. A regulatory role of these miRNAs in trophoblast proliferation, migration and invasion was highlighted and may also be important during equine pregnancy establishment. Transcriptomics studies have shown involvement of DEGs in the counteraction of trophoblast invasion in the horse [14]. Interestingly, miR-144 was also found to alter PGE2 production by regulating PTGS2 in murine granulosa cells [63]. 

To further examine the potential role of miRNAs during MRP in the horse, a vertical analysis was performed, integrating differentially expressed miRNAs, mRNAs and proteins in pregnant vs. cyclic mares. Validated targets for the differentially expressed miRNAs in pregnant vs. cyclic mares present in miRTarBase were compared with differentially expressed genes and proteins (Table 1). Interaction between miRNAs, mRNAs and proteins and involvement in common GO biological processes showed a prominent role in biological regulation (GO:0065007), as well as in developmental process (GO:0032502) and response to stimulus (GO:0008152) (Figure 4 and Figure 5). Coinciding with findings of Klohonatz et al. [24,34], biological adhesion (GO:0022610) was also present, with several molecules involved in focal adhesion and cell adhesion, even though the identity of the factors involved was different. Furthermore, a role in the regulation of integrin binding, as well as in angiogenesis and response to vascular endothelial growth factor was found, as highlighted previously in the context of MRP in the horse [21]. Overall, differential expression of miRNAs in pregnant versus cyclic mares shows involvement in biological processes linked to pregnancy establishment. However, vertical analysis did not identify the unequivocal signal for MRP in the horse. We hypothesized to find upregulation of miRNAs coinciding with downregulation of the target mRNAs and proteins, but the opposite was also found. This indicates a role of other mechanisms besides miRNA regulations, such as proteolysis and post-translational mechanisms, and pleas for further validation of the detected candidate miRNA-mRNA pairs in future studies. Furthermore, miRNA targets in miRTarBase were validated in different species, so further confirmation should be obtained in equine specific assays. While literature on this topic is limited in the horse, most studies only detect a small number of differential miRNAs in pregnant versus cyclic horses with contradictory results depending on the timing and the nature of the tissues [32,34,35]. Overall, it seems unlikely that a single miRNA would be the secret messenger for MRP in the horse.

While the number of differentially expressed miRNAs between pregnant and control mares was limited, substantial differences in miRNA expression were observed at the embryo–maternal interface, highlighting a potentially important role of miRNAs in the embryo–maternal dialogue (Figure 1). Vertical analysis of differential expression of miRNAs and corresponding target mRNAs between the embryo and the pregnant endometrium resulted in 274 miRNA-mRNA pairs. Pathway analysis showed that the main molecular function of these mRNAs was binding (GO:0005488), with two thirds of the molecules involved in this process (Figure 5d). Further categorization into biological processes highlighted a role in biological regulation (GO:0065007), response to stimulus (GO:0050896) and developmental process (GO:0032502). Importance of these pathways in the establishment of pregnancy and clear representation of the miRNA-mRNA pairs indicates a regulatory role of miRNAs in the embryo–maternal dialogue around the timing of signaling of MRP in the horse. As such, a large dataset with valuable reference sequences was established in this study, but the predicted miRNA-mRNA pairs should be confirmed in future studies by targeted validation.

## 4. Materials and Methods 

### 4.1. Sampling

All animal handlings were approved by the Ethical Committee of the Faculty of Veterinary Medicine (EC2013/118) of Ghent University. All methods were performed in accordance with the relevant guidelines and regulations. Sampling was performed as described previously, all samples were collected from the same pregnant and control cycles used for proteomics in Smits et al. [26] Briefly a switch back design was followed with 5 reproductively sound Warmblood mares undergoing two different types of cycles: a pregnant cycle (P) and a cyclic control cycle (C). In this way, the samples were paired using the same mare as its own control for pregnancy and the experimental unit was the mare. The order of P and C cycles was randomly altered for the different mares. Mares displaying uterine oedema and a follicle exceeding 35 mm received 1500 IU hCG intravenously and were either inseminated the next day with fresh semen of the same stallion (P) or left unbred (C). Ovulation was evaluated twice daily by ultrasound. In both groups, sampling was performed 13 days after detection of ovulation. To recover undiluted UF, intra-uterine application of a tampon (OB Mini; Johnson & Johnson, Beerse, Belgium) was performed based upon the method described by Wolf° et al. [64] A double gloved technique was used to avoid vaginal contamination. The tampon was left in the uterus for 10 min, and upon removal, it was placed in a Falcon tube at 4 °C until further processing. Subsequently, the mare’s uterus was flushed with sterile Ringer’s solution by means of a modified endotracheal tube to recover the embryo (P). Finally, an endometrial biopsy was taken and stored immediately at −80 °C.

To process the UF, 1 mL of sterile water (B60, Biosolve, Valkenswaard, The Netherlands) was infused on top of the tampon, and the tampon was attached in the upper part of the Falcon tube by fixing the cord with the cap. Subsequently, the Falcon tube was centrifuged for 20 min at 1000× *g* at 4 °C. The supernatant was collected and stored in aliquots at −80 °C. Meanwhile, the embryo was isolated in a petri dish, and the YS fluid was collected by aspiration with a 21 G needle and stored in aliquots at −80 °C. The capsule was removed from the embryo and stored separately. Finally, the embryonic tissue was also stored separately at −80 °C.

An overview of the experimental design is presented in Figure 1. For miRNA-Sequencing, endometrial biopsies and UF from pregnant and cyclic mares and embryonic tissue (E) and yolk sac fluid (YS) from the pregnant cycles were used. Half of these samples were also used for mRNA-Sequencing, namely the endometrial biopsies from pregnant and cyclic mares and the embryonic tissue. For the other samples, miRNA-Sequencing was combined with proteomics [36].

### 4.2. RNA Extraction

Total RNA was extracted from the UF and the YS fluid samples for miRNA analysis, using the miRNeasy Serum/Plasma kit (Qiagen, Antwerp, Belgium, according to the manufacturers’ instructions. For RNA extraction from the endometrial and the embryonic tissue samples, the RNeasy Mini Kit (Qiagen) was combined with the RNeasy MinElute Cleanup Kit (Qiagen) in order to extract both small RNAs and larger RNAs (>200 nt) separately from the same tissue sample for mRNA-and miRNA-Sequencing respectively.

### 4.3. Messenger-RNA-Sequencing and Data Analysis

After RNA extraction, the concentration and quality of the total extracted RNA were checked by using the ‘Quant-it ribogreen RNA assay’ (Life Technologies, Grand Island, NY, USA) and the RNA 6000 Nano chip (Agilent Technologies, Santa Clara, CA, USA), respectively. Due to low quality, samples from cyclic mare 5 were not used for RNA-Sequencing. Subsequently, 1° µg of RNA was used to perform an Illumina sequencing library preparation using the NEBNext Ultra Directional RNA Library Prep Kit (New England Biolabs, Ipswich, MA, USA) according to the manufacturer’s protocol. During library preparation, 12 PCR cycles were used. Libraries were quantified by qPCR, according to Illumina’s protocol ‘Sequencing Library qPCR Quantification protocol guide’, version February 2011. A High Sensitivity DNA chip (Agilent Technologies) was used to control the library’s size distribution and quality. Sequencing was performed on a high throughput Illumina NextSeq 500 flow cell generating 75° bp single reads.

Per sample, on average 60 ± 16 million reads were generated. First, these reads were trimmed using cutadapt [65] version 1.11 to remove the Illumina adaptor sequence. The trimmed reads were mapped against the equine EquCab 2.80 reference genome using CLC genomics workbench version 9.0.1 (CLC). Count tables were made by counting the reads that mapped against the genes defined by the Ensembl EquCab 2.80 Gene transfer format file and exported from CLC.

Differential gene expression analysis between groups of samples was performed using edgeR [66]. Two differential gene expression analyses were performed. For each separate analysis, the following steps were performed. (1) Reads were normalized using edgeR’s standard normalization method. (2) Only genes with a counts per million (cpm) above 4 in at least four samples were retained. (3) A general linear model was built, and statistical testing was done using the likelihood ratio test. Genes having a false discovery rate (FDR) < 0.05 and a fold change (FC) > 2 were considered significantly differential. All sequences were submitted in the NCBI Gene Expression Omnibus.

### 4.4. MicroRNA-Sequencing and Data Analysis

Total RNA samples were quantified and quality-checked as described for the mRNA Seq samples. MicroRNA sequencing libraries were constructed and size-selected with the TailorMix miRNA Sample Preparation Kit V2 (SeqMatic, Fremont, CA, USA) using 100 ng total RNA and amplified by 18 PCR cycles. Library quality and concentration was determined as described for the mRNA libraries. Sequencing of the miRNA libraries was done as single-read 35 on an Illumina MiSeq. 

Sequencing read quality was checked using FastQC (v1.15). Adapter trimming and removal of reads shorter than 17 bp, having a quality score lower than 20 or containing any ambiguities, was done using cutadapt (v1.11) [65]. Feature counting, identification of known miRNAs and prediction of putative novel miRNA were done using the miRPro pipeline [67]. MicroRNA data from miRBase (release 22) [68] and the corresponding annotated horse genome (EquCab2.0) were used as a reference. All mammalian data in miRBase was used for the prediction of novel miRNA.

Differential expression analyses were done in R using the edgeR (v3.22.3) package [66] and the SARTools (v1.4.1) wrapper [69]. Differentially expressed features were considered statistically significant when having a fold change of at least 2 and an FDR smaller or equal to 0.05. Putative target genes for differentially expressed miRNA were retrieved from miRTarBase (release 7.0) [70]. This database contains miRNA-target interactions which are validated experimentally by reporter assays, western blot, microarray, and next-generation sequencing experiments in different species. Targets present in miRTarBase for the DE miRNAs were subsequently compared with DE mRNAs and proteins found in our study. All sequences were submitted in the NCBI Gene Expression Omnibus (GSE141450).

## 5. Conclusions

In this research, large reference datasets were created, examining expression of miRNAs, mRNAs and proteins at the embryo–maternal interface around the timing of MRP signaling in the horse. Analysis of differential expression showed high conformity with literature on transcriptomics, highlighting differentially expressed genes described in equine pregnancy establishment. Vertical assessment linking differential expression of miRNA, mRNA and proteins confirmed involvement in pregnancy establishment, embryo quality, steroid synthesis and prostaglandin regulation, but the link between differential miRNAs and their targets was limited and did not indicate the identity of an unequivocal signal for MRP in the horse. Differential expression at the embryo–maternal interface was prominent with involvement of miRNA-mRNA target pairs in binding, regulation, signaling and development pathways, highlighting a potential role of miRNAs in embryo–maternal communication during early pregnancy in the horse. These data provide a strong basis for future targeted studies.

## Figures and Tables

**Table 1:** Targets of differentially expressed miRNAs in the endometrial biopsies (B) and uterine fluid (UF) of pregnant (P) versus cyclic (C) mares on day 13 after ovulation. Targets of differentially expressed miRNAs validated in miRTarBase were compared with differentially expressed mRNAs and proteins in the same mares.

**miRNA B**	**miRNA P vs. C** **miRNA P vs. C**	**mRNA B**	**mRNA P vs. C**	**Gene Description**
eca-miR-30c	Down	HDAC4	Down	histone deacetylase 4
eca-miR-30c	Down	DLL4	Down	delta like canonical Notch ligand 4
eca-miR-30c	Down	LRRC8D	Up	leucine rich repeat containing 8 VRAC subunit D
eca-miR-30c	Down	LRRC8B	Up	leucine rich repeat containing 8 VRAC subunit B
eca-miR-130a	Up	ELL2	Down	elongation factor for RNA polymerase II 2
eca-miR-130a	Up	TGFBR1	Down	transforming growth factor beta receptor 1
eca-miR-130a	Up	DLL4	Down	delta like canonical Notch ligand 4
eca-miR-130a	Up	BTBD3	Up	BTB domain containing 3
eca-miR-130a	Up	CDCA4	Up	cell division cycle associated 4
eca-miR-130a	Up	EGLN3	Up	egl-9 family hypoxia inducible factor 3
eca-miR-130a	Up	MET	Up	MET proto-oncogene, receptor tyrosine kinase
eca-miR-130a	Up	LIPA	Up	lipase A, lysosomal acid type
eca-miR-130a	Up	MB21D2	Up	Mab-21 domain containing 2
eca-miR-132	Up	CD55	Up	CD55 molecule (Cromer blood group)
eca-miR-132	Up	GCNT1	Up	glucosaminyl (N-acetyl) transferase 1
eca-miR-144	Down	SLC7A2	Down	solute carrier family 7 member 2
eca-miR-144	Down	ELL2	Down	elongation factor for RNA polymerase II 2
eca-miR-144	Down	SAMD12	Down	sterile alpha motif domain containing 12
eca-miR-144	Down	NR2F2	Up	nuclear receptor subfamily 2 group F member 2
eca-miR-144	Down	MET	Up	MET proto-oncogene, receptor tyrosine kinase
eca-miR-221	Up	FN1	Down	fibronectin 1
eca-miR-221	Up	NHSL1	Down	NHS like 1
eca-miR-221	Up	USP18	Up	ubiquitin specific peptidase 18
eca-miR-221	Up	TNFSF10	Up	TNF superfamily member 10
eca-novel-miR-132	Up	LIPG	Up	lipase G, endothelial type
eca-novel-miR-132	Up	BACE2	Up	beta-secretase 2
eca-novel-miR-146	Up	SPTLC3	Up	serine palmitoyltransferase long chain base subunit 3
**miRNA B**	**miRNA P vs. C**	**Protein UF**	**Protein P vs. C**	**Gene Description**
eca-miR-30c	Down	TKT	Up	Transketolase
eca-miR-30c	Down	RAC1	Up	Rac family small GTPase 1
eca-miR-30c	Down	PFN1	Up	profilin 1
eca-miR-130a	Up	TPP1	Down	tripeptidyl peptidase 1
eca-miR-132	Up	SH3BGRL	Up	SH3 domain-binding glutamic acid-rich-like protein
eca-miR-144	Down	MSN	Down	moesin (membrane-organizing extension spike protein)
eca-miR-144	Down	PLS1	Down	plastin 1
eca-miR-144	Down	RAC1	Up	Rac family small GTPase 1
eca-miR-221	Up	YWHAE	Up	tyrosine 3-monooxygenase/tryptophan 5-monooxygenase activation protein epsilon
eca-miR-221	Up	CLIC1	Up	Chloride intracellular channel protein
eca-miR-221	Up	YWHAB	Up	tyrosine 3-monooxygenase/tryptophan 5-monooxygenase activation protein beta
**miRNA UF**	**miRNA P vs. C**	**mRNA B**	**mRNA P vs. C**	**Gene Description**
eca-miR-23b	Up	CA2	Down	carbonic anhydrase 2
eca-miR-23b	Up	ABCC1	Up	ATP binding cassette subfamily C member 1
eca-miR-23b	Up	MET	Up	MET proto-oncogene, receptor tyrosine kinase
**miRNA UF**	**miRNA P vs. C**	**Protein UF**	**Protein P vs. C**	**Gene Description**
eca-miR-23b	Up	CAP1	Down	adenylyl cyclase-associated protein
eca-miR-23b	Up	NOL11	down	nucleolar protein 11
eca-miR-23b	Up	CCT7	up	chaperonin containing TCP1 subunit 7

## Supplementary Materials

Supplementary materials can be found at https://www.mdpi.com/1422-0067/21/2/419/s1.

## Abbreviations

MRP	Maternal recognition of pregnancy
UF	Uterine fluid
YS	Yolk sac
P	Pregnant
C	Cyclic
DEGs	Differentially expressed genes
DE	Differential expression
